# HSPB7 interacts with dimerized FLNC and its absence results in progressive myopathy in skeletal muscles

**DOI:** 10.1242/jcs.179887

**Published:** 2016-04-15

**Authors:** Liang-Yi Juo, Wern-Chir Liao, Yen-Ling Shih, Bih-Ying Yang, An-Bang Liu, Yu-Ting Yan

**Affiliations:** 1 Institute of Biochemistry and Molecular Biology, National Yang-Ming University, Taipei 112, Taiwan; 2 Institute of Biomedical Sciences, Academia Sinica, Taipei 115, Taiwan; 3 Department of Neurology, Buddhist Tzu Chi General Hospital and Buddhist Tzu Chi University, Hualien 970, Taiwan

**Keywords:** FLNC, HSPB7, Myopathy

## Abstract

HSPB7 belongs to the small heat-shock protein (sHSP) family, and its expression is restricted to cardiac and skeletal muscles from embryonic stages to adulthood. Here, we found that skeletal-muscle-specific ablation of the *HspB7* does not affect myogenesis during embryonic stages to postnatal day 1 (P1), but causes subsequent postnatal death owing to a respiration defect, with progressive myopathy phenotypes in the diaphragm. Deficiency of HSPB7 in the diaphragm muscle resulted in muscle fibrosis, sarcomere disarray and sarcolemma integrity loss. We identified dimerized filamin C (FLNC) as an interacting partner of HSPB7. Immunofluorescence studies demonstrated that the aggregation and mislocalization of FLNC occurred in the muscle of *HspB7* mutant adult mice. Furthermore, the components of dystrophin glycoprotein complex, γ- and δ-sarcoglycan, but not dystrophin, were abnormally upregulated and mislocalized in HSPB7 mutant muscle. Collectively, our findings suggest that HSPB7 is essential for maintaining muscle integrity, which is achieved through its interaction with FLNC, in order to prevent the occurrence and progression of myopathy.

## Abstract (precis)

**Highlighted Article:** HSPB7 plays a crucial role in the maintenance of the muscle integrity, possibly through stabilizing the function of FLNC.

## INTRODUCTION

The cardiovascular heat-shock protein (cvHsp) HSPB7 is a member of the small heat-shock protein (sHSP or HSPB) family (Krief et al., 1999; Vos et al., 2008). To date, the genes of 10 members of the sHSP family have been identified from the human genome. All sHSPs contain a conserved domain called the α-crystallin domain. sHSPs are molecular ATP-independent chaperones that can store aggregated proteins as folding-competent intermediates, conferring enhanced stress resistance to cells by suppressing the aggregation of denaturing proteins (Sun and MacRae, 2005b). Under stress conditions, some sHSPs that are high-molecular-mass complexes dissociate into a small oligomer or a dimer through phosphorylation regulation, preventing the irreversible aggregation of denaturing proteins (Sun and MacRae, 2005a). For example, HSPB7, HSPB8, HSPB6 and HSPB9 can inhibit the aggregation of mutated Huntingtin protein containing different sizes of glutamine repeats (Carra et al., 2005; Vos et al., 2010). Several studies have demonstrated that mammalian sHSPs exhibit the ability to interact and/or modulate the structure and dynamics of the cytoskeleton. HSPB1, HSPB4 and crystallin (CryAB, also known as HSPB5) and HSP20 (also known as HSPB6) can interact with microfilaments and intermediate filaments of the cytoskeleton (Brown et al., 2007; Dreiza et al., 2005; Iwaki et al., 1994; Lavoie et al., 1993, 1995; Liang and MacRae, 1997; Maddala and Rao, 2005; Nicholl and Quinlan, 1994; Perng et al., 1999), and influence their stability and assembly or disassembly, particularly under proteotoxic stress conditions (Iwaki et al., 1994; Lavoie et al., 1993, 1995; Liang and MacRae, 1997; Nicholl and Quinlan, 1994; Perng et al., 1999). HSPB1, HSPB2, HSPB3, HSPB5, HSPB6, HSPB7 and HSPB8 are detected in cardiac and skeletal muscles. Studies have shown that all known mutations in HSPB1, HSPB3 and HSPB8 (Benndorf et al., 2014) are identified in individuals with motor neuropathies, whereas those in HSPB5 can result in desmin-related myopathies (Liu et al., 2006; Simon et al., 2007). Even though the precise molecular and cellular consequences of sHSP mutations are incompletely understood, studies have suggested that sHSPs play crucial roles in maintaining the physiological function of and preventing pathology in striated muscles and motor neurons.

Although HSPB7 is the most potent suppressor of the aggregation of mutated Huntingtin protein (Vos et al., 2010), the HSPB7 gene is one of the most highly expressed genes in the heart (Krief et al., 1999). It has been shown that HSPB7 binds to filamin A (FLNA) and translocates from the cytosol to myofibrils during muscle ischemia (Golenhofen et al., 2004). Overexpression of HSPB7 can reduce the amount of tachypacing-induced F-actin stress fibers upon attenuation of the RhoA GTPase pathway (Ke et al., 2011). Notably, HSPB7 is a potential early biomarker of myocardial infarction and an independent risk factor for acute coronary syndrome (Chiu et al., 2012). Single nucleotide polymorphisms (SNPs) in *HSPB7* have been increasingly associated with cardiomyopathies and heart failure (Cappola et al., 2010; Garnier et al., 2015; Stark et al., 2010). Moreover, studies conducted using the zebrafish model have shown that a reduction of HSPB7 expression disrupts left- and right-axis patterning and affects cardiac morphogenesis (Lahvic et al., 2013; Rosenfeld et al., 2013). Overall, these findings demonstrate the essential role of HSPB7 in cardiac development and functional maintenance.

Proteomics studies have shown that there are drastic increases in HSPB7 expression in the dystrophin-deficient mdx diaphragm and aged skeletal muscle fibers (Doran et al., 2007; Lewis et al., 2009). Such increased expression of stress molecules seems to be a required cellular response during the pathological changes of muscles (Nishimura and Sharp, 2005). However, the importance of this cellular response and the role of HSPB7 in the molecular pathogenesis of muscle abnormalities remain unclear.

Here, we explored the biological and biochemical function of HSPB7 in skeletal muscles *in vivo*. We established an *HspB7* skeletal-muscle-specific knockout mouse model to elucidate the physiological function of HSPB7 in muscles. We found that HSPB7 is not required for sarcomeric assembly in skeletal muscle. However, progressive myopathy phenotypes were observed in adult mutant mice. We identified that the actin-binding protein FLNC interacts with HSPB7, and demonstrated that the absence of HSPB7 results in the disruption of FLNC localization and an increase in FLNC aggregation in muscles. Thus, our results indicate that HSPB7 plays a crucial role in protecting the integrity of the sarcomeric Z-line and sarcolemma to maintain the normal functioning of skeletal muscles.

## RESULTS

### HSPB7 is expressed in striated muscles

**Fig. 1.**
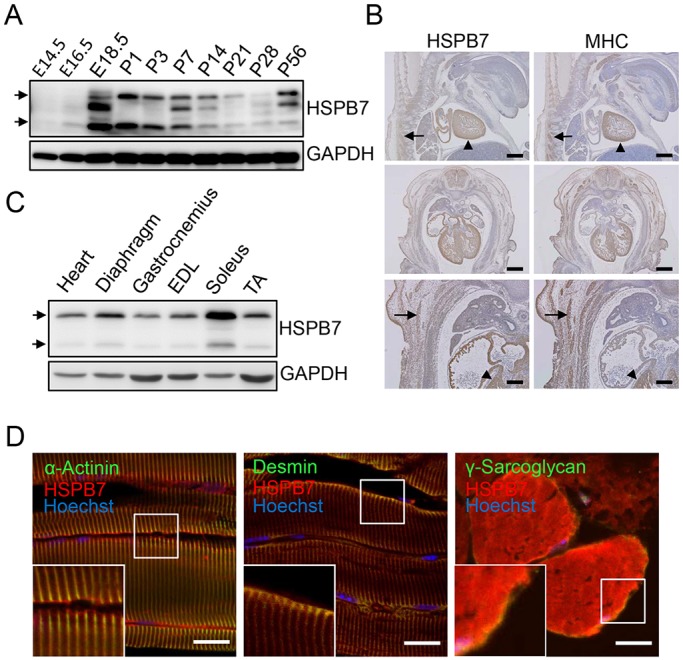
**Expression and localization of HSPB7 in skeletal muscle.** (A) Western blot analysis of calf muscle showing HSPB7 expression from embryonic stages to adulthood (E14.5 to P56). (B) Immunohistochemical analysis of HSPB7 and MHC expression at E13.5 (arrows, muscle tissue; arrowheads, heart). (C) Western blot analysis of HSPB7 expression in the heart and various skeletal muscles. GAPDH was used as loading control. EDL, extensor digitorum longorum; TA, tibialis anterior. (D) Subcellular localization of HSPB7 in the soleus muscle of adult mice. The muscle sections were co-immunostained with antibodies against HSPB7 (red) and desmin (green), sarcomeric α-actinin (green) or γ-sarcoglycan (green). The nucleus was visualized by Hoechst 33342 staining. The arrows in A and C represent the expression of HSPB7. Scale bars: 500 μm (B, upper and middle panel); 200 μm (B, lower panel); 10 μm (D).

We investigated *in vivo* HSPB7 expression in the skeletal muscle from embryonic stages [embryonic day (E) 14.5] to adulthood. Western blotting results showed that HSPB7 was expressed at E14.5 and increased from E16.5 to postnatal day (P)1 and then decreased from P3 to P28. Thereafter, HSPB7 expression increased again at 8 weeks of age (Fig. 1A). Immunoblot analysis identified multiple forms of HSPB7 with different molecular mass (arrows in Fig. 1A,C), suggesting that HSPB7 expression might be regulated by alternative splicing or posttranslational modification. Immunohistochemical staining showed that HSPB7 was not only expressed in the heart but also in the intercostal muscle, in comparison with the myofiber marker protein myosin 1 [MYH1, hereafter referred to as myosin heavy chain (MHC)] (Fig. 1B). These results indicate that HSPB7 is expressed during embryonic primary myogenesis, because primary myofibers are formed in mice from E12 to E14 (Biressi et al., 2007). Western blot analysis showed that HSPB7 expression was higher in the heart, diaphragm and soleus muscles than in the gastrocnemius, tibialis anterior and thigh muscles in adult mice (Fig. 1C). To identify the localization of HSPB7 in skeletal muscle, confocal fluorescence microscopic analysis was performed in longitudinal sections and cross-sections of soleus muscle of adult mice. Co-staining results showed that HSBP7 mainly colocalized with α-actinin, desmin and γ-sarcoglycan (Fig. 1D), indicating that HSPB7 is located at the sarcomeric Z-line and costamere and sarcolemma of the skeletal muscle.

### HSPB7 is upregulated during muscle regeneration and C2C12 myoblast differentiation

To explore whether HSPB7 is involved in skeletal muscle myogenesis, we examined HSPB7 expression during differentiation of C2C12 mouse myoblasts. Through serum restriction, C2C12 myoblasts were induced to differentiate into myotubes and express MyoG and MHC. Immunoblot analysis showed that HSPB7, MyoG and MHC were expressed at the same time during C2C12 differentiation (Fig. S1A). We investigated HSPB7 expression using the mouse tibialis anterior muscle regeneration model. Consistent with the results of C2C12 myoblast differentiation, HSPB7 expression was also upregulated in parallel with MyoD, another myogenesis marker, during muscle regeneration (Fig. S1B). Notably, after BaCl_2_-induced tibialis anterior muscle injury, the expression of HSPB7 and MyoD were both transiently upregulated from day 3 to day 14 during muscle regeneration (Fig. S1B).

### HSPB7 is not required for myogenesis during embryonic stages

To investigate the biological function of HSPB7 in skeletal muscle, *HspB7* conditional knockout (CKO) mice were generated. In addition to engineering a gene targeting vector to contain *loxP* sequences flanking exons 2 and 3 of *HspB7*, we knocked in a FLAG-tag sequence fused to the C-terminal of HSPB7 (Fig. S1C). Southern blot analysis confirmed successful targeting in embryonic stem cells (ESCs) and germline transmission of resulting chimeras (Fig. S1D,E). Despite knocking in the FLAG-tag sequence, *HspB7^Flox/Flox^* mice were healthy and showed normal behavior and exercise activity, and no substantial abnormality was detected in these mice in comparison with wild-type mice. The skeletal muscle CKO offspring genotype ratios did not differ from the expected 1:1:1:1 Mendelian ratio on postnatal day 1 (P1) (Table 1). To confirm that *HspB7* was inactivated in CKO mice, we analyzed HSPB7 expression in muscles including the diaphragm and limb muscles on P1. Four immunoreactive polypeptides, of 11, 12, 17 and 18 kDa, were detected in *HspB7^Flox/+^* muscles upon using the HSPB7 antibody in western blotting (Fig. S1H). The immunoreactive signals from the 11- and 17-kDa polypeptides (gray arrows in Fig. S1H) were expressed from the HSPB7 wild-type allele, and those from the 12- and 18-kDa polypeptides (black arrows in Fig. S1H) were expressed from the Flox allele. None of the four immunoreactive polypeptides could be detected in a sample of muscles from *HspB7* CKO mice, suggesting that the recombinase activity of the *HSA-Cre* transgene almost completely eliminated the *HspB7* Flox allele sequence in muscle tissues. No significant difference in gross morphology (Fig. S1F) and body weight (Fig. S1G) was observed in CKO mice at E18.5 and P1. Moreover, the expression level of sarcomeric protein α-actinin and FLNC in the muscles of CKO mice remained the same as in controls on P1 (Fig. S1H). The results indicate that HSPB7 is not required for myogenesis during the embryonic stage.

**Table 1.**
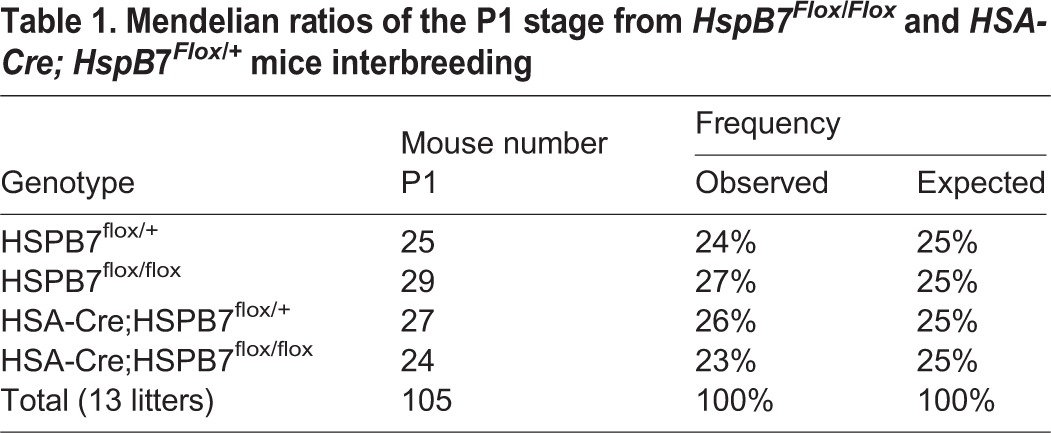
**Mendelian ratios of the P1 stage from *HspB7^Flox/Flox^* and *HSA-Cre; HspB*7*^Flox/+^* mice interbreeding**

### Loss of HSPB7 causes a progressive myopathy phenotype in mice

**Fig. 2.**
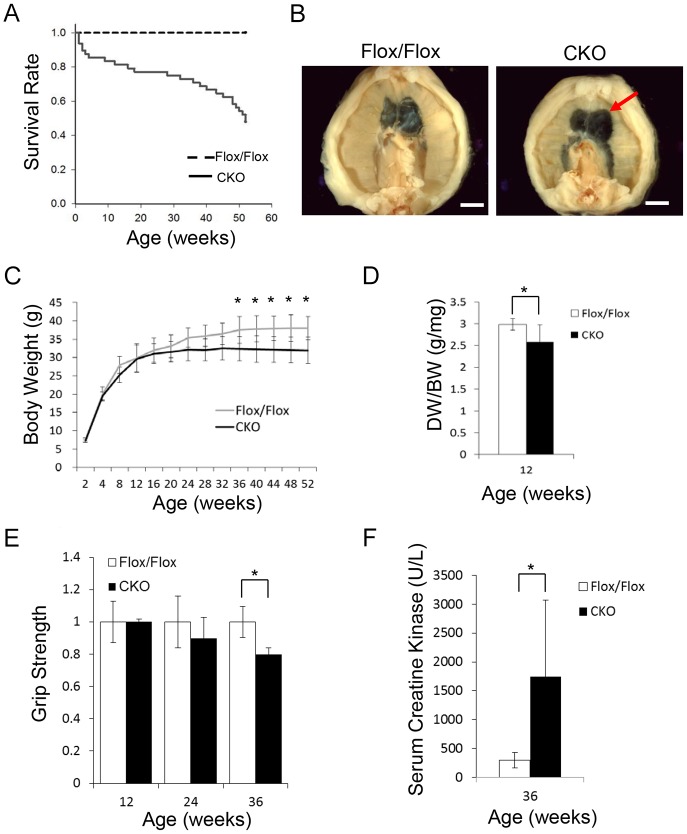
**Characterization of *HspB7* CKO mice.** (A) Survival curve of *HspB7* CKO and control (Flox/Flox) mice. (B) Abdominal view of dissected diaphragms from 12-week-old *HspB7* CKO and control mice. A costal-sided diaphragmatic defect (arrow) was observed in *HspB7* CKO mice. (C) The growth curve of CKO and control mice. Mice of each genotype were weighed every 4 weeks. A significant difference in body weight (BW) was observed between CKO and control mice after the age of 36 weeks (*n*=5; mean±s.e.m.). (D) Significant decreases in diaphragm weight (DW) of 12-week-old CKO mice (*n*=6; mean±s.e.m.). (E) A decrease in forelimb grip strength was observed in CKO mice (*n*=4; mean±s.d.). (F) At 36 weeks of age, a significant increase in serum creatine kinase levels was observed in CKO mice (*n*=8 for CKO and *n*=6 for Flox/Flox; mean±s.d.). Scale bars: 4 mm (B). *P<0.05 (Student's *t*-test).

Although no substantial abnormality was detected in *HspB7* CKO mice at P1, these mice began to die at the age of postnatal 1 week. The survival curve showed a wide spectrum of lethality in *HspB7* CKO mice (Fig. 2A). Anatomical examination showed that *HspB7* CKO mice had a defective diaphragm with reduced muscle mass (arrow in Fig. 2B). The diaphragm muscles of CKO mice were significantly lighter than those of controls at 12 weeks of age (Fig. 2D). A significant decrease in body weight was observed in *HspB7* CKO mice after the age of 40 weeks, but not in young adult mice (Fig. 2C). To determine whether loss of HSPB7 in diaphragm muscles impairs respiratory function, we performed whole-body plethysmography in mice at 12 weeks of age. We found that the respiratory peak expiratory flow and relaxation time were significantly increased in *HspB7* CKO mice at the lower breath frequency. The degree of enhanced pause (Penh) was significantly elevated in *HspB7* CKO mice compared with that in control mice (Table 2); measurement of Penh is commonly used to noninvasively assess respiratory function in mice (Lin, 2001). These results suggest that loss of HSPB7 in diaphragm muscles leads to pathology, impairing respiratory function and resulting in possible lethality. We also measured the grip strength of the forelimbs in CKO mice; no significant change in grip strength was found in young adult mice until the age of 36 weeks (Fig. 2E), suggesting that a progressive myopathy phenotype occurred in mutant mice. In agreement with this result, a significantly increasing percentage of central nuclei in limb muscle fibrils in 36-week-old, but not in young adult, CKO mice could also be detected (Fig. S2A–C). Consistent with this finding, the level of serum creatine kinase in *HspB7* CKO mice was significantly elevated compared to at the same age as in controls (Fig. 2F). Notably, no significant abnormality was detected in the sarcomeric structure by confocal fluorescence microscopic analysis in the soleus muscle of young adult mice (Fig. S2D).

**Table 2.**
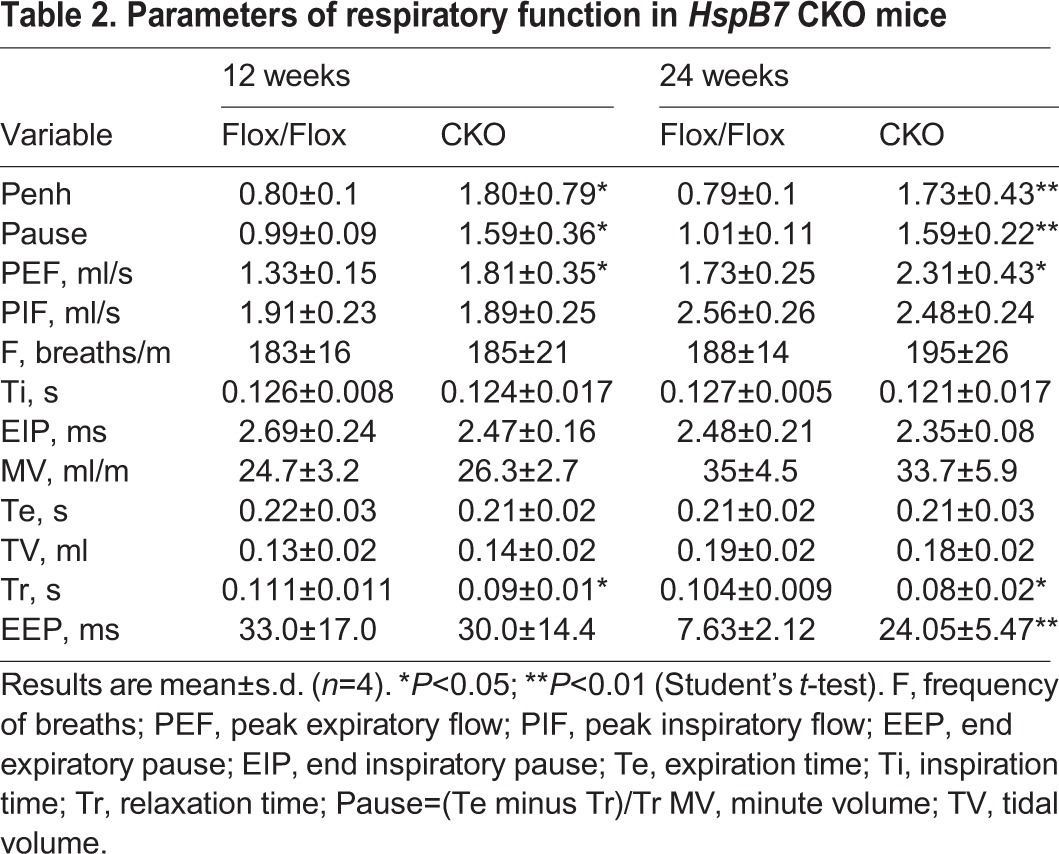
**Parameters of respiratory function in *HspB7* CKO mice**

### Diaphragm muscle defect in *HspB7* CKO mice

**Fig. 3.**
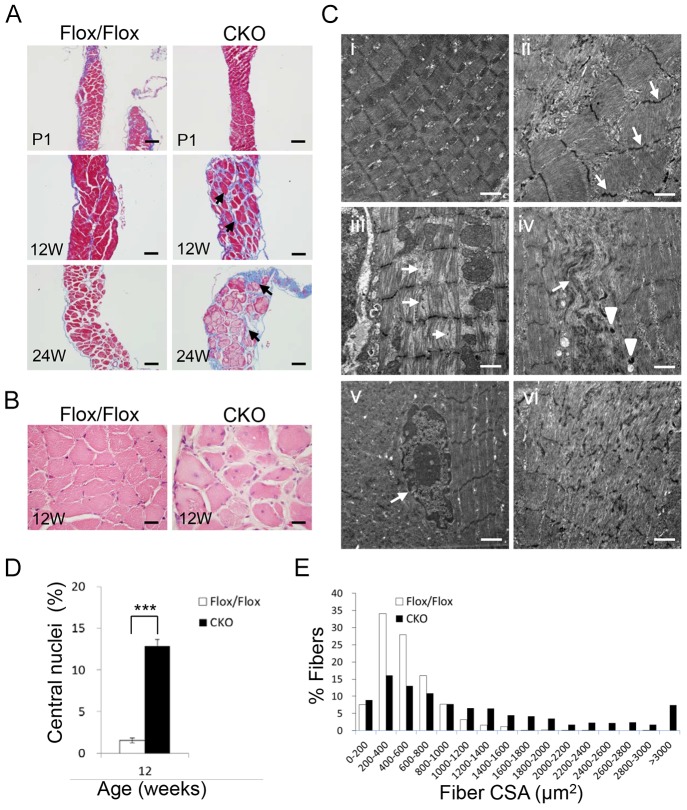
**Diaphragm muscle defects in *HspB7* CKO mice.** (A) Diaphragm muscle sections from control and CKO mice were stained with Masson trichrome. The diaphragm muscle cross-section on P1 revealed no defects in CKO mice. At 12–24 weeks (W) of age, Masson trichrome staining revealed interstitial fibrosis (aniline blue stain). (B) H&E-stained diaphragm histological sections from Flox/Flox and CKO mice at 12 weeks. (C) Transmission electron micrographs (TEMs) of longitudinally sectioned diaphragm muscles from control (Flox/Flox) and CKO mice. (i) Control (Flox/Flox). Z-line streaming (arrows in ii), causing filament disruption, (arrows in iii) and Z-line disruption (vi) were observed. Focal rearrangements (arrows in iv), small rod bodies (arrowheads in iv) and central muscle nuclei (arrow in v) were observed in the diaphragm of CKO mice. (D) Quantification of central nucleation in the diaphragm of control (Flox/Flox) and CKO mice: the number of muscle fibers with central nuclei is represented as a percentage of the total number of muscle fibers analyzed (mean±s.d.; *n*=10 fields, 3 mice per group). (E) Myofiber size (CSA) distribution in the diaphragm muscle of 12-week-old control (Flox/Flox) and CKO mice (*n*=8 fields, 3 mice per group). ***P<0.001 (Student's *t*-test). Scale bars: 100 μm (A); 20 μm (B); 1 μm (C).

Histological examination revealed the progressive defects in the diaphragm muscle. At P1, the diaphragm appeared normal (Fig. 3A). However, morphological defects were detected in the diaphragm of CKO mice by 12–24 weeks of age. Masson trichrome staining revealed a dramatic increase in collagen accumulation in the diaphragm of CKO mutants compared with controls (Fig. 3A). Our results showed that considerable fibrosis occurred in the diaphragm of *HspB7* CKO mice at 12 weeks of age, but not in the soleus muscle (data not shown). Furthermore, an additional histological abnormality was the increase in central nuclei in the diaphragm of CKO mice compared with control mice (Fig. 3B,D), indicating the occurrence of muscle fiber regeneration. In addition, CKO mice tended to have larger muscle fibers (Fig. 3B). A significant increase in cross-sectional area (CSA) was detected in the diaphragm muscle of CKO mice (Fig. 3E).

To examine the defect in the sarcomeric structure in the muscles of *HspB7* CKO mice, muscle ultrastructure was examined under a transmission electron microscope. The sarcomeric myofibers were organized in diaphragm muscles collected from 6-month-old controls, with normal M-lines and Z-lines (Fig. 3Ci). The actin filaments were interdigitated with thick filaments and incorporated into regular sarcomeres appropriately. Sarcomeric filament disarray was frequently observed in the diaphragm muscle of CKO mice. In the mutant diaphragm, focal abnormalities included sarcomeric Z-line streaming (arrows in Fig. 3Cii), the insertion of an additional sarcomere (Fig. 3Cii) and myofibrillar disorganization (Fig. 3Civ,vi) with focal loss of cross-striation. Filament disruption (arrows in Fig. 3Ciii), small rod bodies (arrowheads in Fig. 3Civ), and vacuoles and central nuclei (arrow in Fig. 3Cv) were observed in the diaphragm sections of 24-week-old mice.

### Interaction and subcellular colocalization of HSPB7 with dimerized FLNC

**Fig. 4.**
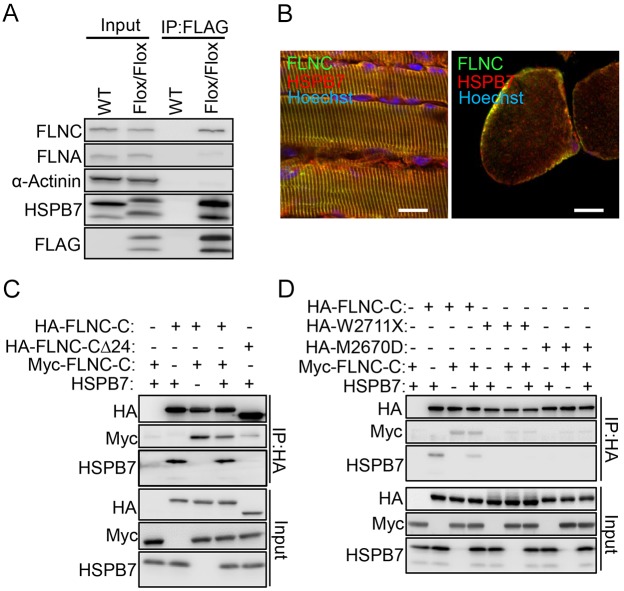
**HSPB7 interacts and colocalizes with FLNC.** (A) Diaphragm muscle from adult *Hspb7^Flox/Flox^* and wild-type (WT) mice was lysed and incubated with anti-FLAG M2 affinity gel for co-immunoprecipitation (IP) of proteins binding to HSPB7, and analyzed by western blotting. (B) The colocalization of HSPB7 and FLNC was assessed by confocal microscopy. The muscle sections were co-immunostained with antibodies against HSPB7 (red) and FLNC (green). The nucleus was visualized by Hoechst 33342 staining. (C) A construct of FLNC lacking the dimerization region (HA–FLNC-CΔ24) was overexpressed in HEK293T cells. The domains mediating interaction between HSPB7 and FLNC were assessed through co-immunoprecipitation *in vitro*. (D) The dimerization region deletion and mutations of FLNC-C (HA–W2711X and HA–M2670D) were overexpressed in HEK293T cells. The domains mediating interaction between HSPB7 and FLNC were assessed through co-immunoprecipitation *in vitro*. Scale bars: 10 μm.

To elucidate the molecular mechanism and functional role of HSPB7, we isolated proteins that interacted with HSPB7 by screening a mouse E10.5 embryonic yeast two-hybrid library. Notably, only filamin family proteins including filamin A, B and C were identified as interacting proteins of HSPB7 (data not shown). The filamins (FLNs) are a family of actin-binding proteins that stabilize cortical three-dimensional F-actin networks and link them to cellular membranes (van der Flier and Sonnenberg, 2001; Zhou et al., 2010). FLNC is expressed specifically in striated and cardiac muscle (Xu et al., 1998). Notably, we also observed that FLNC expression was upregulated, as for HSPB7, during muscle regeneration and C2C12 myoblast differentiation (Fig. S1A,B). We confirmed the interaction between HSPB7 and FLNC by using a co-immunoprecipitation assay (Fig. 4A). In addition, the colocalization of HSPB7 and FLNC at the Z-line of the sarcomere and sarcolemmal membrane in soleus muscles of adult mice was detected using confocal fluorescence microscopic analysis (Fig. 4B).

Next, we examined the interaction between HSPB7 and FLNC in the HEK293T cell culture system. Similar to a report of HSPB7 interacting with the C-terminal of FLNA (Krief et al., 1999), we found that HA–FLNC-C [the immunoglobulin (Ig)-like domains 19–24] interacts with HSPB7 and Myc–FLNC-C, but not the truncated mutant protein Myc–FLNC-CΔ24 (Ig-like domains 19–23) (Fig. 4C). The FLNs exists in dimer form in cells through interactions between Ig-like domain 24 (Pudas et al., 2005). The FLNC mutant protein W2710X (with a 8130G→A mutation resulting in a tryptophan at position 2710 becoming a stop codon; W2710X) contains a truncated FLNC Ig-like domain 24 without the dimerization activity, meaning that the protein is prone to self-protein aggregation (Vorgerd et al., 2005). However, the interaction of FLNC W2710X with actin and sarcoglycans is not affected (Lowe et al., 2007). In addition, the point mutation, M2669D, has also been reported to disrupt the dimerization of FLNC Ig-like domain 24 (Pudas et al., 2005). In co-immunoprecipitation analysis, we found that this mutant FLNC protein lost the ability not only to form the dimer but also to interact with HSPB7 (Fig. 4D), indicating that HSPB7 interacts with the dimerized Ig-like domain 24 of FLNC. Moreover, our results showed that the presence of HSPB7 did not affect the efficiency of FLNC dimerization (Fig. 4D).

### Loss of HSPB7 results in FLNC aggregation and sarcolemmal fragility

**Fig. 5.**
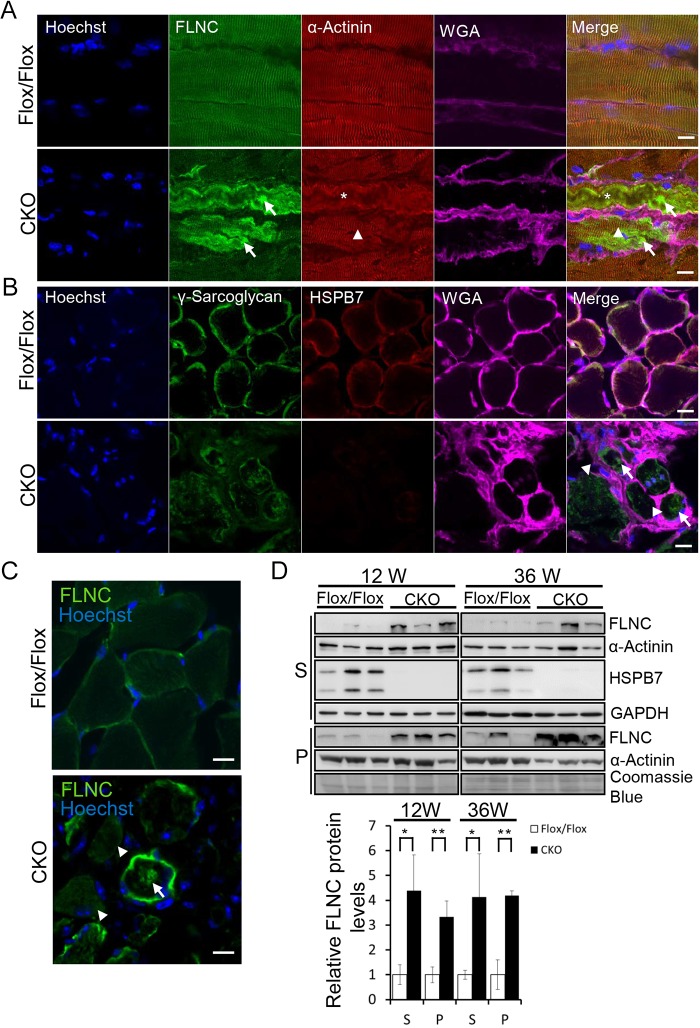
**Loss of HSPB7 results in FLNC and γ-sarcoglycan protein aggregation.** Confocal micrographs of longitudinal sections (A) and cross-sections (B,C) of the diaphragm of 12-week-old mice. Specific antibodies were used to identify the distributions of the sarcomeric components α-actinin (Z-line), FLNC and γ-sarcoglycan as indicated. FLNC (arrows in A,C) and γ-sarcoglycan (arrows in B) aggregation were observed in the diaphragm muscle. The arrangement of α-actinin was normal (arrowheads in A). However, Z-line disarray was also observed in the diaphragm of CKO mice (asterisks in A). FLNC and γ-sarcoglycan were discontinuous or missing in some regions of the sarcolemma (arrowheads in B,C). Extracellular matrix accumulation was labeled with wheat germ agglutinin (WGA). FLNC mislocalizes on the sarcolemma (arrowheads in C). The nucleus was visualized by Hoechst 33342 staining. (D) Western blot analysis of expression levels of FLNC and α-actinin in the diaphragm muscle. The muscle homogenate supernatant (S) and pellet (P) fractions from control (Flox/Flox) and CKO mice at 12 and 36 weeks of age. GAPDH was used to verify the loading amount in the supernatant. Coomassie Blue staining (45–75 kDa) was used to verify the loading amount in the pellet. The graph shows the mean±s.d. densitometry results (*n*=3). *P<0.05; **P<0.01 (Student's *t*-test). Scale bars: 10 μm (A); 50 μm (B); 20 µm (C)

Because FLNC interacts with HSPB7, we further examined the effect of HSPB7 absence on FLNC expression in mice. First, confocal fluorescence microscopic analysis showed that the immunoreactivity of FLNC was significantly increased in diaphragm longitudinal section in *HspB7* CKO mice (arrow in Fig. 5A), but not in control mice. Notably, impaired distributions of FLNC (Fig. 5A), γ-sarcoglycan (Fig. 5B) and δ-sarcoglycan (Fig. S3A) and an accumulation of extracellular matrix (WGA staining in Fig. 5A,B and Fig. S3A) were observed in the cross-section of the diaphragm muscle in *HspB7* CKO mice. The arrangement of α-actinin was also disrupted in some abnormal myofibrils (asterisks in Fig. 5A). However, staining revealed FLNC aggregation in myofibrils with a normal α-actinin pattern (arrowheads in Fig. 5A) or disarray pattern (arrows in Fig. 5A). The amount of FLNC aggregation in diaphragm was significantly higher than in gastrocnemius and soleus muscle (Fig. S3B). In accordance with the results of FLNC staining in the diaphragm of CKO mice, immunoblot analysis showed an upregulation of FLNC in the homogenate supernatant and pellet fractions from the diaphragm in mice at 12 and 36 weeks of age (Fig. 5D), and from the soleus muscle at 36 weeks of age (Fig. S2E). Furthermore, FLNC aggregation was also observed in the tibialis anterior muscle during muscle regeneration in *HspB7* CKO but not control mice (Fig. S2F). The aggregation of FLNC in *HspB7* CKO muscle might be caused by the upregulation of FLNC in regenerated muscles (Figs S1A, S2G). To test whether the FLNC aggregation phenotype specifically occurred in *HspB7* mutant muscles, we examined the dystrophin-deficient mdx mice. In the diaphragm of mdx mice, FLNC was increased at the sarcolemma. Comparing with the staining of the *HspB7* mutant diaphragm, only few muscle fibers were observed with protein aggregation of FLNC (Fig. S4). Some previous studies have also reported that the expression and membrane localization of FLNC is increased in mdx muscles (Thompson et al., 2000; Zhang et al., 2007). Next, we tested whether HSPB7 suppressed the aggregation of FLNC in a cell culture system, given that HSPB7 is the most potent polyQ aggregation suppressor. However, overexpression of HSPB7 in HEK293T cells does not affect aggregation of the FLNC C-terminal protein (Fig. S3C), suggesting that HSPB7 does not maintain the biochemical activity of FLNC by suppressing FLNC aggregation.

We further examined whether the impaired expression of FLNC and γ-sarcoglycan affects the muscle, because both are required for maintaining the structural integrity of cardiac and skeletal muscles (Fujita et al., 2012). Evans Blue Dye (EBD) was intraperitoneally injected, and membrane integrity was subsequently evaluated through microscopic visualization of EBD uptake by muscle fibers after 48 h. Analysis of diaphragm muscles showed a higher number of EBD-positive fibers in muscles of *HspB7* CKO mice compared with those of controls (Fig. S3F,G). The loss of HSPB7 resulted in pronounced muscle weakness and progressive myopathological defects because of abnormal localization and irreversible aggregation of FLNC and sarcoglycans.

## DISCUSSION

In this study, we demonstrated the expression and subcellular location of HSPB7 in muscles from embryonic stages to adulthood. We found that HSPB7 expression increased during muscle regeneration and C2C12 myoblast differentiation. We also established a muscle-specific knockout mouse model to explore the biological function of HSPB7. We provided compelling evidence indicating that the loss of HSPB7 function is sufficient to induce the development of a progressive myopathy phenotype associated with myofibrillar disorganization and sarcolemma disruption in mice. Specifically, we found that HSPB7 deficiency *in vivo* is associated with (1) defects in sarcolemma integrity with accumulation of intersarcomeric and extracellular aggregated proteins, (2) development of myofibrillar disarray and decreased respiration activity in the diaphragm, and (3) decreased survival rates, which are associated with age-dependent muscular damage. We identified FLNC as a protein that interacted with HSPB7, and found that the expression of FLNC and HSPB7 colocalized in muscle cells. Furthermore, we demonstrated that HSPB7 interacts only with dimerized FLNC, and that its absence causes the abnormal localization and aggregation of FLNC. FLNC expression is restricted to striated muscles, as is HSPB7, and it interacts with many muscle proteins involved in muscle formation and function. *Flnc* mutant mice have severely reduced birth weight, with fewer muscle fibers and primary myotubes, indicating defects in primary myogenesis (Dalkilic et al., 2006). In addition, *Flnc* neonatal mutants also die early with respiration defects. Despite the less-severe phenotype, *HspB7* CKO mutant mice showed a progressive myopathy phenotype with the aggregation and mislocalization of its interacting protein FLNC. Interestingly, the amount of FLNC aggregation correlated with the severity of myopathy phenotype in *HspB7* CKO muscle. These observations suggest that HSPB7 is vital for achieving the full biochemical and biological activity of FLNC.

FLNC localizes at the periphery of the Z-line and crosslinks thin filaments at their ends in the myofibrillar Z-line. It has been suggested that FLNC acts as a mechanosensor to detect mechanical strain and transmit mechanical stimuli by undergoing conformational changes (Razinia et al., 2012; Ulbricht et al., 2013). Abnormal localization and expression of FLNC have been detected in numerous myopathies (Bonnemann et al., 2003; Sewry et al., 2002). In limbgirdle muscular dystrophies (LGMDs) and Duchenne muscular dystrophy (DMD) patients, several Z-disc-associated proteins and sarcolemmal proteins, including FLNC and sarcoglycans, are mislocalized and form intracellular aggregates (Thompson et al., 2000). Consistent with these findings, an increasing expression, aggregation and mislocalization of FLNC and sarcoglycans was observed in the muscles of *HspB7* CKO postnatal mice. Any perturbation in the distribution of these proteins caused by loss of HSPB7 might destabilize the muscle cell membrane and weaken the sarcomeric structure, leading to muscular defects.

FLNC is a γ-sarcoglycan- and δ-sarcoglycan-interacting protein (Thompson et al., 2000). Abnormal localization and expression of γ- and δ-sarcoglycan were observed in the *HspB7* CKO diaphragm, but the localization of dystrophin appeared normal. The γ- and δ-sarcoglycans are members of dystrophin glycoprotein complex (DGC), which acts to mechanically stabilize interactions between the cytoskeleton, membrane and extracellular matrix. It has been reported that loss of γ-sarcoglycan leads to muscle membrane defects, but dystrophin remains localized at the plasma membrane and does not require sarcoglycan for normal distribution (Hack et al., 1998). The cause of muscle membrane integrity loss in the absence of HSPB7 might not only be due to abnormal localization of FLNC but also to that of γ- and δ-sarcoglycan. The role of HSPB7 in FLNC and sarcoglycan regulation requires further study. Interestingly, HSPB7 does not function as a chaperone molecule to directly prevent FLNC aggregation. These findings suggest that HSPB7 might play a chaperone-independent role to enable FLNC to resist the conformational changes in response to mechanical strain. In agreement with our finding, another sHsp family member CryAB [HSPB5, also known as l(2)efl in *Drosophila*] has also been identified to be required for Z-band patterning and might maintain myofibrillar integrity through interacting with Cheerio (the *Drosophila* homolog of human filamin) in *Drosophila* muscles (Wojtowicz et al., 2015). However, the function of HSPB7 in maintaining the mouse muscle integrity might specifically stabilize the interaction of FLNC with DGC, given that sarcomere structure is less affected in mutant skeletal muscles except for the diaphragm. These results also suggest a possibility that the different sHSPs might play a cooperative effect in maintaining the full activity of filamin protein.

Recent studies have shown that a chaperone-assisted selective autophagy (CASA) machinery incorporates tension sensing, autophagosome formation and transcription regulation to maintain the filamin protein homeostasis in mammalian cells (Arndt et al., 2010; Ulbricht et al., 2013). Moreover, BAG3 can bind to LATS1 or LATS2 and AMOTL1 or AMOTL2, which abrogates the cytoplasmic retention of YAP1 and WWTR1 and thereby induces filamin transcription to maintain actin anchoring and crosslinking under mechanical strain (Ulbricht et al., 2013). Indeed, an upregulation of FLNC was observed in the HspB7 mutant muscle. Loss of HSPB7 might directly or indirectly cause FLNC unfolding or conformational changes that would further activate the CASA pathway, thereby leading to FLNC transcription stimulation.

The most obvious features of *HspB7* CKO mice were the reduced diaphragm muscle mass, loss of muscle fibers and severe alterations in muscle morphology in postnatal life. These were also the major causes of postnatal lethality in *HspB7* CKO mice, because the diaphragm is the key muscle for respiration. The molecular mechanism that caused the diaphragm to degenerate faster than other muscles in HSPB7-deficient mice remains unclear. One possible explanation is that high contraction activity combined with a less-stable structure of the mechanosensor caused by the absence of HSPB7 could contribute to the more pronounced deleterious changes observed for this muscle *in vivo*.

HSPB7 is a co-chaperone protein mainly expressed in striated muscle and, to a lesser extent, is upregulated in dystrophin-deficient mdx diaphragm, aged skeletal muscles and damaged heart. Because only some SNPs of *HspB7* have thus far been reported to be associated with heart-failure-related diseases, how crucial a role HSPB7 plays in muscle physiology is unclear. This is the first comprehensive study revealing information regarding the biological function of HSPB7 in muscle. The approaches of this study have revealed that HSPB7 plays a crucial role in the maintenance of the myofiber structure, possibly through stabilizing the function of FLNC. Our findings warrant future investigation into the functional roles of HSPB7 in regulating the dimeric form of FLNC. Moreover, we provided a new animal model to study myofibril myopathy.

## MATERIALS AND METHODS

### Cell culture and differentiation

Mouse C2C12 myoblasts were grown in Dulbecco's modified Eagle's medium (DMEM, Gibco), supplemented with 10% fetal bovine serum (FBS) and 100 U/ml of penicillin and 100 μg/ml of streptomycin. Cells were maintained in an incubator at 37°C in 5% CO_2_. Cell differentiation was induced by culturing 90% confluent cells in differentiation medium (DMEM, with 2% horse serum). The medium was changed daily. Differentiation was assessed morphologically by the appearance of multinucleated myotubes. The cells were washed in PBS and lysed with lysis buffer for western blot analysis.

HEK293T cells were transfected with HSPB7 and FLNC by using TurboFect (Thermo Scientific). The cells were washed in PBS and lysed with 1× cell lysis buffer (catalog no. 9803, Cell Signaling Technology) with complete protease inhibitor cocktail (catalog no. 11836145001, Roche Applied Sciences). The insoluble fraction was collected through centrifugation at 10,000 ***g*** for 15 min, and an equal volume of 2× sodium dodecyl sulfate-polyacrylamide gel electrophoresis (SDS-PAGE) sample buffer was then added to the supernatants and insoluble fraction for western blot analysis. The cells were lysed in RIPA buffer for co-immunoprecipitation experiments.

### Muscle regeneration

Injury was induced in the tibialis anterior muscle of adult mice through injection with BaCl_2_ (50 μl of 1.2% BaCl_2_), as previously described (Caldwell et al., 1990). Mice were killed at defined periods following injury (24 h, 48 h, 3 days, 5 days, 7 days, and 14 days), and tibialis anterior muscles were lysed for western blot analysis or fixed overnight in 4% paraformaldehyde for paraffin-embedded sectioning. All animal experiments were performed in accordance with the guidelines of the Institutional Animal Care and Use Committee of IBMS, Academia Sinica.

### Establishment of *HspB7* CKO allele

To generate *HspB7* CKO animals, the Cre-loxp recombination system was used, in which HSPB7 exons 2 and 3 were flanked by loxp sites. A gene-targeting vector was generated in which exons 2 and 3 of the *HspB7* gene were flanked by loxP sites, and a FLAG-tag sequence was inserted before the *HspB7* stop codon. A Sac I site was also inserted into exon 3. The neomycin resistance (Neo) cassette was flanked by two Flp recognition target (FRT) recombination sites and an inserted XbaI site. After homologous recombination, blastocyst injection of targeted embryonic stem cells was used to obtain mice with the targeted allele. Chimeric mice were crossed with C57BL/6 mice to obtain germline transmission. Mice analyzed in this study were backcrossed to C57BL/6 for four generations. The backcrosses involved mice constitutively expressing the FLP recombinase to remove the neomycin cassette from the targeted allele (Rodríguez et al., 2000) and mice expressing the Cre recombinase under the HSA promoter.

### Generation of HSPB7 CKO mice

For the generation of skeletal-muscle-specific *HspB7* CKO mutants, human α-skeletal actin (HSA)-Cre79 transgenic mice (Miniou et al., 1999) carrying the Cre gene driven by the HSA promoter were bred with *HSPB7^Flox/Flox^* mice onto a mixed 129×C57BL/6 genetic background. Mice were killed or collected at the indicated age for experiments. Cre-negative littermates were used as controls.

### Preparation of tissue extracts and western blotting

Muscle tissue dissected from anesthetized mice was rinsed in cold phosphate-buffered saline (PBS), blotted dry, weighed and then homogenized using TissueLyser LT (Qiagen) in 1× cell lysis buffer (cat#9803, Cell Signaling Technology) with complete protease inhibitor cocktail (catalog no. 11836145001, Roche Applied Sciences). The insoluble fraction was sedimented through centrifugation at 10,000 ***g*** for 15 min, and an equal volume of 2× sodium dodecyl sulfate-polyacrylamide gel electrophoresis (SDS-PAGE) sample buffer was then added to the supernatants and the insoluble fraction (pellet). After heating at 100°C for 10 min, aliquots of the supernatants were stored at −80°C until use. For western blotting experiments, protein extracts (20 μg of total protein) were separated on 8% or 15% polyacrylamide gels and blotted onto polyvinylidene difluoride (PVDF) membranes (Millipore Corporation). Membranes were blocked with blocking buffer (5% nonfat dry milk, 10 mM Tris-HCl, pH 7.6, 150 mM NaCl, and 0.1% Tween 20) and incubated with primary antibodies at 4°C overnight. After incubation with horseradish-peroxidase-conjugated secondary antibodies, antibody binding was detected using enhanced chemiluminescence reagents (Millipore Corporation). Levels of the protein glyceraldehyde-3-phosphate dehydrogenase (GAPDH) were used to normalize the results. The primary antibodies used were: mouse monoclonal anti-α-actinin (clone EA-53, Sigma-Aldrich; 1:1000), anti-GAPDH (clone 6C5, Millipore Corporation; 1:3000), anti-MHC [MF20, Developmental Studies Hybridoma Bank (DSHB), 1:1000], anti-myogenin (anti-MyoG; F5D, DSHB; 1:1000) antibodies; rabbit polyclonal anti-FLAG (F7425, Sigma-Aldrich; 1:3000), anti-Myc (LTK BioLaboratories; 1:3000), anti-HA (Abcam; 1:3000), anti-γ-sarcoglycan (H-60, Santa Cruz Biotechnology; 1:200), anti-MyoD (M-318, Santa Cruz Biotechnology; 1:1000) and anti-dystrophin (ab15277, Abcam; 1:1000) antibodies; goat polyclonal anti-FLNC (K-18, Santa Cruz Biotechnology; 1:1000) antibodies and guinea pig polyclonal antibody against the NH2-terminal of the murine HSPB7 peptide (amino acids 29–84, LTK BioLaboratories).

### Histopathology

Mouse muscle tissues were collected, fixed with 10% formalin buffered with phosphate, and embedded in paraffin. Tissue sections (5 μm) were subjected to hematoxylin and eosin (H&E) and Masson trichrome staining according to standard procedures (Young et al., 2006). For *in vivo* tests of sarcolemma integrity, 12-week-old mice were intraperitoneally injected with 0.1 mg/g body weight Evans Blue Dye (EBD; Sigma) for 2 consecutive days. These mice were euthanized after 18 h, diaphragm and soleus tissues were collected, and EBD-positive myofibers were observed under a stereomicroscope (SMZ1500, Nikon).

### Immunofluorescence and immunohistochemical analysis

For immunofluorescence staining, soleus and diaphragm tissues were isolated from *HspB7* control and CKO mice, directly embedded in optimal cutting temperature compound (OCT), and cryosectioned into 14-μm sections. The sections were postfixed in 2% paraformaldehyde, blocked with 2% bovine serum albumin (BSA), and incubated with the aforementioned primary antibodies at 4°C overnight. After washing in PBS, sections were incubated with secondary antibodies, including FITC- or rhodamine-conjugated goat anti-mouse and anti-rabbit IgG, FITC-conjugated donkey anti-goat IgG, rhodamine-conjugated donkey anti-guinea-pig IgG (Jackson ImmunoResearch Laboratories). Sections were counterstained with 0.5 μg/ml Hoechst 33342 (Cell Signaling Technology). Fluorescence was visualized using a Zeiss LSM700 confocal microscope.

For immunohistochemical analysis, tissue samples were fixed overnight in 4% paraformaldehyde for paraffin-embedded sectioning (5-μm sections). Muscle sections were soaked in antigen retrieval buffer containing 10 mM sodium citrate (pH 6.0) and heated separately in boiling water bath for 40 min. These sections were then incubated with primary antibody against HSPB7 and MHC and horseradish peroxidase (HRP)-conjugated secondary antibody. Antibody binding was detected using diaminobenzidine tetrahydroxychloride (DAB) solution (Jackson ImmunoResearch Laboratories). Subsequently, these sections were counterstained with hematoxylin solution.

### Transmission electron microscopy

Mouse soleus and diaphragm tissues were cut into small blocks in a fixative mixture of glutaraldehyde (1.5%) and paraformaldehyde (1.5%) in phosphate buffer (pH 7.3), as described previously (Wu et al., 2012). Ultrathin sections were cut, mounted, poststained, and observed under a FEI TECNAI G2 F20 S-TWIN electron microscope (Electron Microscope Core Facility of Institute of Cellular and Organismic Biology, Academia Sinica).

### Respiratory function

Respiratory function was measured using Unrestrained Whole-Body Plethysmography (Buxco). Unanesthetized mice were placed into a plethysmograph chamber and allowed to acclimate. Baseline averages of the breathing frequency, peak inspiratory height (PIF), peak expiratory height (PEF), expiratory time (Te), and time to expire 65% of the volume (Rt) were recorded for 10 min. The respiration resistance (Penh), which represents the resistance of the respiratory tract, was calculated using the following formula: Penh=(REF/PIF)×(Te/Rt−1). The data were collected for 90 min. The data were collected for 10 min when the breathing frequency continued to be less than 250 (breaths/min) for 10 min.

### Grip strength

Muscle strength of the forelimbs was assessed using MK-380CM/R (Muromachi Kikai Co., Ltd.). The peak gripping force at the point of grip failure was recorded as grip strength. For each mouse, grip strength was measured in five trials with 30 s of rest between each trial. All grip strength values obtained were normalized to mouse body weight.

### Plasmid construction

A full-length mouse HSPB7 cDNA construct was subcloned into the expression vector pcDNA3. A FLNC C-terminal cDNA wild-type (G2133-P2726) construct was subcloned into the pcDNA3.0-HA and pcDNA3.0C-Myc expression vectors. Constructs with deletion of FLNC-C Ig-like domain 24 for production of FLNC-CΔ24 (amino acid G2133-S2588), W2711X (amino acid G2133-W2711) and M2670D (amino acid G2133-P2726) mutant proteins were subcloned into the pcDNA3.0-HA vector. After successful cloning, the *Flnc* cDNA expression construct was transiently transfected into HEK-293T cells for immunoprecipitation experiments.

### Co-immunoprecipitation experiments

For immunoprecipitation experiments, the diaphragm muscles of adult C57BL/6 mice were lysed in cell lysis buffer (Cell Signaling Technology) with a complete protease inhibitor cocktail (Roche Applied Sciences). Diaphragm extracts (500 μg) from *Hspb7*^Flox/Flox^ and wild-type mice were incubated with the anti-FLAG M2 affinity gel (A2220, Sigma) at 4°C overnight. The beads were washed with lysis buffer and analyzed by western blotting. HEK293T cells were transfected with HA–FLNC expression constructs individually. After 24 h, cells were harvested and co-immunoprecipited using EZview Red Anti-HA affinity gel (E6779, Sigma) according to the manufacturer's protocol.

### Evans Blue Dye injection and immunofluorescence staining

The mdx mice (C57BL/10ScSn-mdx/J; Jackson Laboratory) and CKO mice were injected intraperitoneally with Evans Blue Dye (1 mg/g of body weight). At 2 days after injection, mice were killed, and the diaphragm muscles were collected for subsequent experiments. Diaphragm tissues were isolated from *HspB7* control and CKO mice, directly embedded in optimal cutting temperature compound (OCT), and cryosectioned into 14-μm sections. The sections were postfixed with 50% methanol and 50% acetone mix, blocked with 2% bovine serum albumin (BSA), and incubated with anti-vinculin (V9131, Sigma; 1:200) antibodies at 4°C overnight. Fluorescence was visualized using a Zeiss LSM700 confocal microscope.

### Statistics

Results are presented as mean±s.d. Comparisons between the two groups were performed using a two-tailed Student's *t*-test. Mouse survival rates were calculated using the Kaplan–Meier method. *P*<0.05 was considered significant when analyzing statistical differences between the different groups of mice.

## References

[JCS179887C1] Arndt V., Dick N., Tawo R., Dreiseidler M., Wenzel D., Hesse M., Fürst D. O., Saftig P., Saint R., Fleischmann B. K. et al. (2010). Chaperone-assisted selective autophagy is essential for muscle maintenance. *Curr. Biol.* 20, 143-148. 10.1016/j.cub.2009.11.022 20060297

[JCS179887C2] Benndorf R., Martin J. L., Kosakovsky Pond S. L. and Wertheim J. O. (2014). Neuropathy- and myopathy-associated mutations in human small heat shock proteins: characteristics and evolutionary history of the mutation sites. *Mutat. Res. Rev. Mutat. Res.* 761, 15-30. 10.1016/j.mrrev.2014.02.004 PMC4157968 24607769

[JCS179887C3] Biressi S., Molinaro M. and Cossu G. (2007). Cellular heterogeneity during vertebrate skeletal muscle development. *Dev. Biol.* 308, 281-293. 10.1016/j.ydbio.2007.06.006 17612520

[JCS179887C4] Bonnemann C. G., Thompson T. G., van der Ven P. F. M., Goebel H. H., Warlo I., Vollmers B., Reimann J., Herms J., Gautel M., Takada F. et al. (2003). Filamin C accumulation is a strong but nonspecific immunohistochemical marker of core formation in muscle. *J. Neurol. Sci.* 206, 71-78. 10.1016/S0022-510X(02)00341-6 12480088

[JCS179887C5] Brown Z., Ponce A., Lampi K., Hancock L. and Takemoto L. (2007). Differential binding of mutant (R116C) and wildtype alphaA crystallin to actin. *Curr. Eye Res.* 32, 1051-1054. 10.1080/02713680701769989 18085469 PMC2425674

[JCS179887C6] Caldwell C. J., Mattey D. L. and Weller R. O. (1990). Role of the basement membrane in the regeneration of skeletal muscle. *Neuropathol. Appl. Neurobiol.* 16, 225-238. 10.1111/j.1365-2990.1990.tb01159.x 2402330

[JCS179887C7] Cappola T. P., Li M., He J., Ky B., Gilmore J., Qu L., Keating B., Reilly M., Kim C. E., Glessner J. et al. (2010). Common variants in HSPB7 and FRMD4B associated with advanced heart failure. *Circ. Cardiovasc. Genet.* 3, 147-154. 10.1161/CIRCGENETICS.109.898395 20124441 PMC2957840

[JCS179887C8] Carra S., Sivilotti M., Chavez Zobel A. T., Lambert H. and Landry J. (2005). HspB8, a small heat shock protein mutated in human neuromuscular disorders, has in vivo chaperone activity in cultured cells. *Hum. Mol. Genet.* 14, 1659-1669. 10.1093/hmg/ddi174 15879436

[JCS179887C9] Chiu T.-F., Li C.-H., Chen C.-C., Chen C.-H., Cheng C.-J., Yan Y.-T. and Yang R.-B. (2012). Association of plasma concentration of small heat shock protein B7 with acute coronary syndrome. *Circ. J.* 76, 2226-2233. 10.1253/circj.CJ-12-0238 22785082

[JCS179887C10] Dalkilic I., Schienda J., Thompson T. G. and Kunkel L. M. (2006). Loss of FilaminC (FLNc) results in severe defects in myogenesis and myotube structure. *Mol. Cell. Biol.* 26, 6522-6534. 10.1128/MCB.00243-06 16914736 PMC1592847

[JCS179887C11] Doran P., Gannon J., O'Connell K. and Ohlendieck K. (2007). Aging skeletal muscle shows a drastic increase in the small heat shock proteins alphaB-crystallin/HspB5 and cvHsp/HspB7. *Eur. J. Cell Biol.* 86, 629-640. 10.1016/j.ejcb.2007.07.003 17761354

[JCS179887C12] Dreiza C. M., Brophy C. M., Komalavilas P., Furnish E. J., Joshi L., Pallero M. A., Murphy-Ullrich J. E., von Rechenberg M., Ho Y. S., Richardson B. et al. (2005). Transducible heat shock protein 20 (HSP20) phosphopeptide alters cytoskeletal dynamics. *FASEB J.* 19, 261-263. 15598710 10.1096/fj.04-2911fje

[JCS179887C13] Fujita M., Mitsuhashi H., Isogai S., Nakata T., Kawakami A., Nonaka I., Noguchi S., Hayashi Y. K., Nishino I. and Kudo A. (2012). Filamin C plays an essential role in the maintenance of the structural integrity of cardiac and skeletal muscles, revealed by the medaka mutant zacro. *Dev. Biol.* 361, 79-89. 10.1016/j.ydbio.2011.10.008 22020047

[JCS179887C14] Garnier S., Hengstenberg C., Lamblin N., Dubourg O., De Groote P., Fauchier L., Trochu J.-N., Arbustini E., Esslinger U., Barton P. J. et al. (2015). Involvement of BAG3 and HSPB7 loci in various etiologies of systolic heart failure: results of a European collaboration assembling more than 2000 patients. *Int. J. Cardiol.* 189, 105-107. 10.1016/j.ijcard.2015.04.003 25889438

[JCS179887C15] Golenhofen N., Perng M. D., Quinlan R. A. and Drenckhahn D. (2004). Comparison of the small heat shock proteins alphaB-crystallin, MKBP, HSP25, HSP20, and cvHSP in heart and skeletal muscle. *Histochem. Cell Biol.* 122, 415-425. 10.1007/s00418-004-0711-z 15480735

[JCS179887C16] Hack A. A., Ly C. T., Jiang F., Clendenin C. J., Sigrist K. S., Wollmann R. L. and McNally E. M. (1998). Gamma-sarcoglycan deficiency leads to muscle membrane defects and apoptosis independent of dystrophin. *J. Cell Biol.* 142, 1279-1287. 10.1083/jcb.142.5.1279 9732288 PMC2149352

[JCS179887C17] Iwaki T., Iwaki A., Tateishi J. and Goldman J. E. (1994). Sense and antisense modification of glial alpha B-crystallin production results in alterations of stress fiber formation and thermoresistance. *J. Cell Biol.* 125, 1385-1393. 10.1083/jcb.125.6.1385 8207065 PMC2290922

[JCS179887C18] Ke L., Meijering R. A. M., Hoogstra-Berends F., Mackovicova K., Vos M. J., Van Gelder I. C., Henning R. H., Kampinga H. H. and Brundel B. J. J. M. (2011). HSPB1, HSPB6, HSPB7 and HSPB8 protect against RhoA GTPase-induced remodeling in tachypaced atrial myocytes. *PLoS ONE* 6, e20395 10.1371/journal.pone.0020395 21731611 PMC3123278

[JCS179887C19] Krief S., Faivre J.-F., Robert P., Le Douarin B., Brument-Larignon N., Lefrere I., Bouzyk M. M., Anderson K. M., Greller L. D., Tobin F. L. et al. (1999). Identification and characterization of cvHsp: a novel human small stress protein selectively expressed in cardiovascular and insulin-sensitive tissues. *J. Biol. Chem.* 274, 36592-36600. 10.1074/jbc.274.51.36592 10593960

[JCS179887C20] Lahvic J. L., Ji Y., Marin P., Zuflacht J. P., Springel M. W., Wosen J. E., Davis L., Hutson L. D., Amack J. D. and Marvin M. J. (2013). Small heat shock proteins are necessary for heart migration and laterality determination in zebrafish. *Dev. Biol.* 384, 166-180. 10.1016/j.ydbio.2013.10.009 24140541 PMC3924900

[JCS179887C21] Lavoie J. N., Gingras-Breton G., Tanguay R. M. and Landry J. (1993). Induction of Chinese hamster HSP27 gene expression in mouse cells confers resistance to heat shock. HSP27 stabilization of the microfilament organization. *J. Biol. Chem.* 268, 3420-3429. 8429018

[JCS179887C22] Lavoie J. N., Lambert H., Hickey E., Weber L. A. and Landry J. (1995). Modulation of cellular thermoresistance and actin filament stability accompanies phosphorylation-induced changes in the oligomeric structure of heat shock protein 27. *Mol. Cell. Biol.* 15, 505-516. 10.1128/MCB.15.1.505 7799959 PMC232001

[JCS179887C23] Lewis C., Carberry S. and Ohlendieck K. (2009). Proteomic profiling of x-linked muscular dystrophy. *J. Muscle Res. Cell Motil.* 30, 267-279. 10.1007/s10974-009-9197-6 20082121

[JCS179887C24] Liang P. and MacRae T. H. (1997). Molecular chaperones and the cytoskeleton. *J. Cell Sci.* 110, 1431-1440. 9224761 10.1242/jcs.110.13.1431

[JCS179887C25] Lin C.-C. (2001). Noninvasive method to measure airway obstruction in nonanesthetized allergen-sensitized and challenged mice. *Respiration* 68, 178-185. 10.1159/000050489 11287833

[JCS179887C26] Liu Y., Zhang X., Luo L., Wu M., Zeng R., Cheng G., Hu B., Liu B., Liang J. J. and Shang F. (2006). A novel alphaB-crystallin mutation associated with autosomal dominant congenital lamellar cataract. *Invest. Ophthalmol. Vis. Sci.* 47, 1069-1075. 10.1167/iovs.05-1004 16505043 PMC2078606

[JCS179887C27] Lowe T., Kley R. A., van der Ven P. F. M., Himmel M., Huebner A., Vorgerd M. and Furst D. O. (2007). The pathomechanism of filaminopathy: altered biochemical properties explain the cellular phenotype of a protein aggregation myopathy. *Hum. Mol. Genet.* 16, 1351-1358. 10.1093/hmg/ddm085 17412757

[JCS179887C28] Maddala R. and Rao V. P. (2005). alpha-Crystallin localizes to the leading edges of migrating lens epithelial cells. *Exp. Cell Res.* 306, 203-215. 10.1016/j.yexcr.2005.01.026 15878345

[JCS179887C29] Miniou P., Tiziano D., Frugier T., Roblot N., Le Meur M. and Melki J. (1999). Gene targeting restricted to mouse striated muscle lineage. *Nucleic Acids Res.* 27, e27 10.1093/nar/27.19.e27 10481039 PMC148637

[JCS179887C30] Nicholl I. D. and Quinlan R. A. (1994). Chaperone activity of alpha-crystallins modulates intermediate filament assembly. *EMBO J.* 13, 945-953. 7906647 10.1002/j.1460-2075.1994.tb06339.x PMC394896

[JCS179887C31] Nishimura R. N. and Sharp F. R. (2005). Heat shock proteins and neuromuscular disease. *Muscle Nerve* 32, 693-709. 10.1002/mus.20373 15962334

[JCS179887C32] Perng M. D., Cairns L., van den I. P., Prescott A., Hutcheson A. M. and Quinlan R. A. (1999). Intermediate filament interactions can be altered by HSP27 and alphaB-crystallin. *J. Cell Sci.* 112, 2099-2112. 10362540 10.1242/jcs.112.13.2099

[JCS179887C33] Pudas R., Kiema T.-R., Butler P. J. G., Stewart M. and Ylänne J. (2005). Structural basis for vertebrate filamin dimerization. *Structure* 13, 111-119. 10.1016/j.str.2004.10.014 15642266

[JCS179887C34] Razinia Z., Mäkelä T., Ylänne J. and Calderwood D. A. (2012). Filamins in mechanosensing and signaling. *Annu. Rev. Biophys.* 41, 227-246. 10.1146/annurev-biophys-050511-102252 22404683 PMC5508560

[JCS179887C35] Rodríguez C. I., Buchholz F., Galloway J., Sequerra R., Kasper J., Ayala R., Stewart A. F. and Dymecki S. M. (2000). High-efficiency deleter mice show that FLPe is an alternative to Cre-loxP. *Nat. Genet.* 25, 139-140. 10.1038/75973 10835623

[JCS179887C36] Rosenfeld G. E., Mercer E. J., Mason C. E. and Evans T. (2013). Small heat shock proteins Hspb7 and Hspb12 regulate early steps of cardiac morphogenesis. *Dev. Biol.* 381, 389-400. 10.1016/j.ydbio.2013.06.025 23850773 PMC3777613

[JCS179887C37] Sewry C. A., Müller C., Davis M., Dwyer J. S. M., Dove J., Evans G., Schröder R., Fürst D., Helliwell T., Laing N. et al. (2002). The spectrum of pathology in central core disease. *Neuromuscul. Disord.* 12, 930-938. 10.1016/S0960-8966(02)00135-9 12467748

[JCS179887C38] Simon S., Michiel M., Skouri-Panet F., Lechaire J. P., Vicart P. and Tardieu A. (2007). Residue R120 is essential for the quaternary structure and functional integrity of human alphaB-crystallin. *Biochemistry* 46, 9605-9614. 10.1021/bi7003125 17655279

[JCS179887C39] Stark K., Esslinger U. B., Reinhard W., Petrov G., Winkler T., Komajda M., Isnard R., Charron P., Villard E., Cambien F. et al. (2010). Genetic association study identifies HSPB7 as a risk gene for idiopathic dilated cardiomyopathy. *PLoS Genet.* 6, e1001167 10.1371/journal.pgen.1001167 20975947 PMC2958814

[JCS179887C40] Sun Y. and MacRae T. H. (2005a). Small heat shock proteins: molecular structure and chaperone function. *Cell. Mol. Life Sci.* 62, 2460-2476. 10.1007/s00018-005-5190-4 16143830 PMC11138385

[JCS179887C41] Sun Y. and MacRae T. H. (2005b). The small heat shock proteins and their role in human disease. *FEBS J.* 272, 2613-2627. 10.1111/j.1742-4658.2005.04708.x 15943797

[JCS179887C42] Thompson T. G., Chan Y.-M., Hack A. A., Brosius M., Rajala M., Lidov H. G. W., McNally E. M., Watkins S. and Kunkel L. M. (2000). Filamin 2 (FLN2): a muscle-specific sarcoglycan interacting protein. *J. Cell Biol.* 148, 115-126. 10.1083/jcb.148.1.115 10629222 PMC3207142

[JCS179887C43] Ulbricht A., Eppler F. J., Tapia V. E., van der Ven P. F. M., Hampe N., Hersch N., Vakeel P., Stadel D., Haas A., Saftig P. et al. (2013). Cellular mechanotransduction relies on tension-induced and chaperone-assisted autophagy. *Curr. Biol.* 23, 430-435. 10.1016/j.cub.2013.01.064 23434281

[JCS179887C44] van der Flier A. and Sonnenberg A. (2001). Structural and functional aspects of filamins. *Biochim. Biophys. Acta* 1538, 99-117. 10.1016/S0167-4889(01)00072-6 11336782

[JCS179887C45] Vorgerd M., van der Ven P. F. M., Bruchertseifer V., Löwe T., Kley R. A., Schröder R., Lochmüller H., Himmel M., Koehler K., Fürst D. O. et al. (2005). A mutation in the dimerization domain of filamin c causes a novel type of autosomal dominant myofibrillar myopathy. *Am. J. Hum. Genet.* 77, 297-304. 10.1086/431959 15929027 PMC1224531

[JCS179887C46] Vos M. J., Hageman J., Carra S. and Kampinga H. H. (2008). Structural and functional diversities between members of the human HSPB, HSPH, HSPA, and DNAJ chaperone families. *Biochemistry* 47, 7001-7011. 10.1021/bi800639z 18557634

[JCS179887C47] Vos M. J., Zijlstra M. P., Kanon B., van Waarde-Verhagen M. A. W. H., Brunt E. R. P., Oosterveld-Hut H. M. J., Carra S., Sibon O. C. M. and Kampinga H. H. (2010). HSPB7 is the most potent polyQ aggregation suppressor within the HSPB family of molecular chaperones. *Hum. Mol. Genet.* 19, 4677-4693. 10.1093/hmg/ddq398 20843828

[JCS179887C48] Wojtowicz I., Jablonska J., Zmojdzian M., Taghli-Lamallem O., Renaud Y., Junion G., Daczewska M., Huelsmann S., Jagla K. and Jagla T. (2015). Drosophila small heat shock protein CryAB ensures structural integrity of developing muscles, and proper muscle and heart performance. *Development* 142, 994-1005. 10.1242/dev.115352 25715399

[JCS179887C49] Wu C.-Y., Chen Y.-F., Wang C.-H., Kao C.-H., Zhuang H.-W., Chen C.-C., Chen L.-K., Kirby R., Wei Y.-H., Tsai S.-F. et al. (2012). A persistent level of Cisd2 extends healthy lifespan and delays aging in mice. *Hum. Mol. Genet.* 21, 3956-3968. 10.1093/hmg/dds210 22661501

[JCS179887C50] Xu W., Xie Z., Chung D. W. and Davie E. W. (1998). A novel human actin-binding protein homologue that binds to platelet glycoprotein Ibalpha. *Blood* 92, 1268-1276. 9694715

[JCS179887C51] Young B., Lowe J. S., Stevens A., Heath J. W., Deakin P. J., Woodford P. and O'Dowd G. (2006). *Wheater's Functional Histology: A Text and Colour Atlas*, pp. 448 Elsevier Health Sciences.

[JCS179887C52] Zhang M., Liu J., Cheng A., DeYoung S. M. and Saltiel A. R. (2007). Identification of CAP as a costameric protein that interacts with filamin C. *Mol. Biol. Cell* 18, 4731-4740. 10.1091/mbc.E07-06-0628 17898075 PMC2096606

[JCS179887C53] Zhou A.-X., Hartwig J. H. and Akyürek L. M. (2010). Filamins in cell signaling, transcription and organ development. *Trends Cell Biol.* 20, 113-123. 10.1016/j.tcb.2009.12.001 20061151

